# Superconductivity of thulium substituted clathrate hexahydrides at moderate pressure

**DOI:** 10.1038/s41598-024-61400-z

**Published:** 2024-05-10

**Authors:** Hongyu Huang, Chao Deng, Hao Song, Mingyang Du, Defang Duan, Yanhui Liu, Tian Cui

**Affiliations:** 1 School of Physical Science and Technology, Institute of High Pressure Physics, Ningbo University, Ningbo, 315211 People’s Republic of China; 2 College of Physics, Jilin University, Changchun, 130012 People’s Republic of China

**Keywords:** Hydrides, High pressure, Superconductivity, First principles calculation, Superconducting properties and materials, Superconducting properties and materials

## Abstract

Due to the BCS theory, hydrogen, the lightest element, would be the prospect of room-temperature superconductor after metallization, but because of the difficulty of the hydrogen metallization, the theory about hydrogen pre-compression was proposed that the hydrogen-rich compounds could be a great option for the high *T*_*c*_ superconductors. The superior properties of TmH_6_, YbH_6_ and LuH_6_ indicated the magnificent potential of heavy rare earth elements for low-pressure stability. Here, we designed XTmH_12_ (X = Y, Yb, Lu, and La) to obtain higher *T*_*c*_ while maintaining low pressure stability. Most prominently, YbTmH_12_ can stabilize at a pressure of 60 GPa. Compared with binary TmH_6_ hydride, its *T*_*c*_ was increased to 48 K. The results provide an effective method for the rational design of moderate pressure stabilized hydride superconductors.

## Introduction

Since Kamerlingh Onnes discovered that mercury (Hg) suddenly starts carrying a current without resistance at an extremely low temperature in 1911^1,2^, the achievement of room temperature superconductor is a dream for the superconductivity research. The theory that hydrogen can be metallized at high pressure was developed in 1935 and was proposed by Winger and Huntington^3^. According to the theory of superconductivity proposed by Bardeen, Cooper and Schrieffer in 1957, the transition temperature of superconductivity is proportional to the Debye temperature^4^. Due to this theory, hydrogen, the lightest element, would be the prospect of room-temperature superconductor after metallization^5^, but because of the difficulty of the hydrogen metallization^6,7^, the theory about hydrogen pre-compression was proposed by Ashcroft that the hydrogen-rich compounds could be a great option for the high *T*_*c*_ superconductors^8,9^. The theory of chemical pre-compression refers to the addition of other elements to the synthesized hydrogen-rich compounds at a lower pressure than synthesizing pure hydrogen^10^. Based on this conclusion, many great hydrogen-rich compounds have been designed and predicted to be potential superconductors with high *T*_*c*_^11–13^. The first successful predictions were H_3_S and LaH_10_ with high *T*_*c*_ exceeding 200 K^14–16^, and these predictions were successfully confirmed by experiment soon^17–20^.

Over these years, with the efforts of our researchers, almost all binary hydrides were explored, people commence the study of ternary hydride formed by adding a new element into binary hydrides. In 2019, Li_2_MgH_16_ with the highest *T*_*c*_ to date (473 K at 250 GPa), designed by filling the anti-bonding orbital of the H_2_ molecular unit of MgH_16_ with the element Li^21^. H–C–S compounds and Lu–N–H compounds have been widely studied for some time due to the claimed observation of room temperature superconductivity. However, there are still some controversial issues about the stoichiometry and the crystal structure^22–25^. Recently, a new kind of fluorite-type clathrate ternary hydrides AXH_8_ (A = Ca, Sr, Y, La, X = B, Be, Al) in the main chain of hydrogen alloys has been predicted^26^. The most prominent, LaBeH8, is dynamically stable down to 20 GPa and has a high *T*_*c*_ up to 185 K. The exciting thing is that the cubic clathrate superhydrides La_x_Y_1-x_H_6,10_ have been experimentally synthesized by laser heating of yttrium-lanthanum alloys, which exhibited a maximum critical temperature *T*_*c*_ of 253 K without increasing pressure^27^. According this experiment, it is practicable to incorporate a metal element in the clathrate hydride to keep the compounds steadily.

It is a widespread attention about the prominent superconductivity of the clathrate hydrides. Clathrate hexahydrides *Im*-3* m*-XH_6_ (X = Mg, Ca, Sc, Y, La, Tm, Yb, Lu) are widespread in alkaline earth and rare earth metal superhydrides^16,28–32^. In this structure, there is a body-centered cube (bcc) with center occupied by a metal atom, and there is a H_24_ cage of hydrogen atoms in the void of the bcc lattice. CaH_6_ and YH_6_ have been experimentally synthesized with high *T*_*c*_s of 215 K at 172 GPa^33,34^ and 227 K at 166 GPa, respectively^35^. Theoretically predicted *T*_*c*_s of MgH_6_, ScH_6_ and LaH_6_ are 260 K at 300 GPa, 147 K at 285 GPa and174 K at 100 GPa, respectively. YbH_6_ and LuH_6_ in full *4f.*-orbital shells are predicted to exhibit high *T*_*c*_ superconductivity at relatively low pressures (145 K, 70 GPa vs. 273 K, 100 GPa, respectively)^32^. With unfilled *4f.* orbitals, TmH_6_ is stable at 50 GPa, but has a relatively low *T*_*c*_ at 25 K. There was a report that the structures of superhydrides at low pressure could keep stable by *f* electrons, such as lanthanide clathrate hydrides CeH_9_^36^, PrH_9_^37^ and NdH_9_^38^. Although the filling of the metal atoms’ *f* orbital could make the structure more stable at low pressure, according to current research results, the *T*_*c*_s of hydrides with unfilled *4f.* orbitals are mostly very low.

The properties of TmH_6_, YbH_6_ and LuH_6_ indicated the magnificent potential of such structures for low-pressure stability. In alkaline earth and rare earth metals hydrides *Im*-3* m*-XH_6_ are common, such as CaH_6_^28^, MgH_6_^29^, YH_6_^15,16,30^, ScH_6_^31^, (Tm/Yb/Lu)H_6_^32^. The structure can also be extended into the ternary structure *Pm*-3* m*-ABH_12_, such as (Y,Ca)H_6_^39–41^, (Mg,Ca)H_6_^42^, (Sc,Ca)H_6_^43^, (La,Y)H_6_^44^, (Ca/Sc/Y,Yb/Lu)H_6_^45^. In recent years, based on this sodalite-like clathrate structure, we have designed a series of high-temperature superconductors that can be stable under moderate pressures by adding heavy rare earth elements Yb/Lu to sodalite-like clathrate hydrides^45^. Among them, Y_3_LuH_24_ and YLu_3_H_24_ are the room-temperature superconductors with the lowest stabilizing pressure predicted by current theory (283 K, 120 GPa and 288 K, 110 GPa, respectively). This result shows that room-temperature superconductivity of hydrogen-based superconductors is possible at medium pressure.

In this work, we designed XTmH_12_ (X = Y, Yb, Lu, and La) to obtain higher *T*_*c*_ while maintaining low pressure stability. Most prominently, YbTmH_12_ can stabilize at 60 GPa. Compared with binary TmH_6_ hydride, its *T*_*c*_ was increased to 48 K. The results provide an effective method for the rational design of moderate pressure stabilized hydride superconductors.

## Results

**Figure 1:**
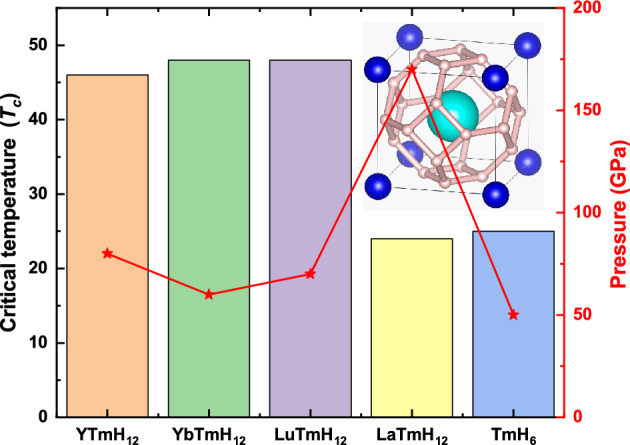
Crystal structures of *Pm*-3* m*-XTmH_12_ at 150 GPa, superconducting critical temperature *T*_*c*_ dynamically stability of compounds XTmH_12_ (X = Y, Yb, Lu and La).

First, we designed a series of ternary clathrate hydrides YTmH_12_ based on the sodalite-like clathrate structure YLuH_12_^45^. The crystal structure of *Pm*-3* m*-YLuH_12_ is shown in Fig. 1. The atoms Y, Tm, and H occupy the 1*b* (0.5, 0.5, 0.5), 1*a* (0, 0, 0), and 12 h (0.25322, 0, 0) Wyckoff positions in the crystal structure. In this structure, there are a bcc lattice of metal atoms and a H_24_ cage which is formed by the hydrogen atom occupying all the tetrahedral void of the lattices, as shown in Fig. 1, and the H_24_ cage is formed by six H-square and eight H-hexagon rings, with two classes of unequal H atoms. In many alkaline earth metals and rare earth metal hydrides there are this kind of structure consisting of metal atom and H_24_ cage. There are two H_2_ accepting electrons from the central metal atoms to form an H_4_ unit, which serves as the cornerstone for the construction of a three-dimensional sodalite gabion and thus makes the structure stable. This unique structure partially occupies the degenerate orbit at the center of the region. The resulting dynamic Jahn–Teller effect contributes to enhanced electron–phonon coupling and leads to high *T*_*c*_ superconductivity.

As is well-known, the impact of the electron correlation effects is particularly significant for 4*f*. systems. In our previous work, we calculated the equation of state (EOS) for YbH_2_ and compared it with the experimental EOS to assess the reliability of our DFT calculations^32^. One can see that there is a good agreement between the theory and experiment for the high-pressure phase *P*6_3_/*mmc* of YbH_2_. The authors of previous work concerned with ytterbium hydrides^46^, used a Hubbard U = 5 eV for lower pressure phases and U = 0 eV for high-pressure phases to reproduce available experimental data, in clear agreement with our results. Therefore, in this work, we select GGA and U = 0 eV for calculation of 4*f* systems.

Next, we try to extend YTmH_12_ to more compounds. In the designed XTmH_12_ structure, at least one of the “pre-compressor” metal atoms is heavy rare earth element Tm, and the other element has a similar radius with Tm, including Na, K, Mg, Ca, Sr, Sc, Y, Yb, Lu, La. Then we calculated the phonon dispersion for all possible components in the pressure range of 50–200 GPa. The stability of the replaced structure is reflected in Fig. 1, we determined that only Y, Yb, Lu and La can stabilize dynamically this ternary sodalite-like clathrate structure.

**Figure 2:**
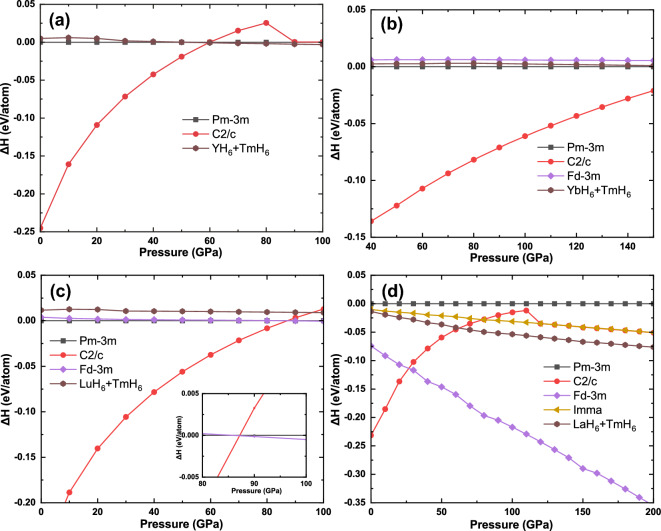
Calculated enthalpies per (**a**) YTmH_12_, (**b**) YbTmH_12_, (**c**) LuTmH_12_, (**d**) LaTmH_12_ as the function of pressure.

To determine the thermodynamic stability of these structures, we performed structure searches at a pressure of 100–200 GPa, focusing on XTmH_12_ (X = Y, Yb, Lu and La) compositions with 1 to 2 formula units. As shown in Fig. 2, None of the structures *Pm*-3* m*-XTmH_12_ have the lowest enthalpy values, which means they are all metastable phases. The enthalpy of *Pm*-3* m*-YTmH_12_ and *Pm*-3* m*-LaTmH_12_ are higher than that of binary hydrides YH_6_ + TmH_6_ and LaH_6_ + TmH_6_, respectively. *Pm*-3* m*-YbTmH_12_ and *Pm*-3* m*-LuTmH_12_ are stable compared to the binaries YbH_6_ + TmH_6_ and LuH_6_ + TmH_6_, respectively, but their enthalpy are higher than that of the other ternary hydrides, such as *C*2/*c*-YbTmH_12_ and *Fd*-3* m*-LuTmH_12_. This means that some difficulties need to be overcome in the experiment to synthesize these structures. However, metastable stable phases can also be synthesized experimentally and even dominate over thermodynamically stable phases^47,48^.

**Figure 3:**
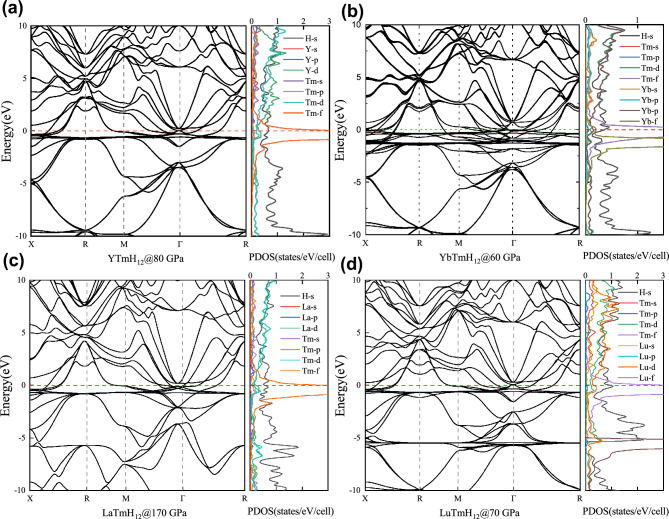
Calculated electronic band structures and projected density of states for (**a**) YTmH_12_, (**b**) YbTmH_12_, (**c**) LuTmH_12_, (**d**) LaTmH_12_.

We calculated their electronic band structures and projected density of states (PDOS). It can be clearly seen that the DOS value of the s electron of H near the Fermi surface is higher than that of the p and d electrons of the metal element. This is because H does not exist in molecular form, but forms a H_24_ cage. However, compared to other high-*T*_*c*_ hydrides with H_24_ cage, such as CaH_6_, YH_6_, and LuH_6_, the DOS values of H's s electrons near the Fermi plane in these structures are not high enough, which is not good news for searching for high-temperature superconductors in hydrides. Furthermore, it is worth mentioning that the DOS of XTmH_12_ has extremely high peaks near the Fermi surface. This is mainly due to the 4*f* orbitals from heavy rare earth elements form a set of localized and almost non-dispersive bands in XTmH_12_. These bands will appear in different positions depending on the outermost electrons of the element. The bands from Tm atom with unfilled 4*f* orbitals appear at the Fermi level in YTmH_12_ (see Fig. 3a), and the bands from Yb atom with full-filled 4*f* orbitals appear about 1 eV below the Fermi level (see Fig. 3b). The bands from Tm atom with unfilled 4*f* orbitals also appear at the Fermi level in LaTmH_12_ (see Fig. 3c), this means that the species of the other metal element has almost no effect on the energy level at which the *4f* electron appears. The bands from Lu atom appear about 6 eV below the Fermi level (see Fig. 3d) because of full-filled 4*f* orbitals and an extra 5d electron. The *f* electrons can enhance the chemical compression effects from metallic elements, helping to stabilize the structure at lower pressures.

To make the prediction more reliable, evaluation of the impact of the electron correlation effects is desired. Therefore, we calculated the band structure using U = 5 eV to figure out how Hubbard-U may modify the band structure (see Fig. S1 in Supplementary Material). After considering U = 5 eV, one flat band is lifted up into the unoccupied regime. This means that the occupation of 4*f* states is changed, that could have substantial impact on the electron–phonon coupling physics. Future studies will focus on the impact of U to pairing strength.

## Discussion

Then, we have compared the electronic density of states at the Fermi level (N_Ef_) in YTmH_12_, YbTmH_12_, LuTmH_12_ and LaTmH_12_ and binary hexahydrides, including YH_6_, TmH_6_, YbH_6_, LuH_6_, LaH_6_, as shown in Table 1. Benefit by *4f* electrons from heavy rare earth elements Tm, large electronic density of states at the Fermi level in the XTmH_12_ is observed, much higher than that of the binary hexahydrides. The large H-derived electronic density of states at the Fermi level is beneficial for strong electron–phonon coupling (EPC) parameter λ. Generally speaking, N_Ef_ indicates all of the candidate electrons to form Cooper pairs. It is clear that the large N_Ef_ plays a positive role in enhancing the EPC λ. However, from the aspect of partial DOS, the contribution from H to the electronic density of states at the Fermi level in XTmH_12_ is not higher than the cases in binary hexahydrides. This suggests that the *4f* electrons will play no role in superconductivity. The contrasting EPC λ in these clathrate hexahydrides is mainly attributed to the disparate intensity of H electrons interacting with optic phonons, rather than the contributions from global electronic structures. Papaconstantopoulos et al. apply the Gaspari-Gyorffy theory to determine that, in CaH_6_, the acoustic modes associated with Ca contribute only 7% to the total value of λ, in contrast to the optic modes associated with hydrogen which contribute 93% for the H^49^. And in LaH_10_, La has only a 2% contribution^50^.

**Table 1:** The calculated electron–phonon coupling (EPC) parameter λ, logarithmic average phonon frequency ω_log_, electron density of states at the Fermi level (N_Ef_, states/spin/Ry/cell), angular momentum components of the DOS at the Fermi level, superconducting critical temperature *T*_*c*_ for compounds YTmH_12_, YbTmH_12_, LuTmH_12_ and LaTmH_12_ and binary hexahydrides, including YH_6_, TmH_6_, YbH_6_, LuH_6_, LaH_6_.

Compounds	Pressure (GPa)	λ	ω_log_ (K)	N_Ef_	States/spin/Ry/cell					*T*_c_ (K)
					X-d	X-f	Tm-d	Tm-f	H–s	
YTmH_12_	80	1.09	583	32.4	2.1	0	1.3	24.6	4.4	40–46
YbTmH_12_	60	1.04	657	37.6	1.2	1.5	0.7	31.6	2.6	42–48
LuTmH_12_	70	1.10	596	32.4	2.2	0	1.4	22.6	6.2	42–48
LaTmH_12_	170	0.79	530	30.2	1.7	0	1.2	23.0	4.3	19–24
YH_6_	120	3.06	829	4.7	2.0	0	–	–	2.2	251–264^30^
TmH_6_	50	0.72	612	29.6	–	–	0.7	27.4	1.3	19–25^32^
YbH_6_	70	2.22	652	8.4	1.0	4.3	–	–	2.7	121–131^32^
LuH_6_	100	3.60	751	4.8	0.1	0	–	–	4.5	227–243^32^
LaH_6_	100	1.83	1244	4.5	0.8	0	–	–	3.6	156–174^16^

The calculated *T*_*c*_s by the Allen-Dynes modified McMillan equation^51^ are shown in Table 1. LaTmH_12_ has a *T*_*c*_ of 19–24 K at 170 GPa. This is not only much higher than the minimum stabilization pressure of 50 GPa for TmH_6_, but also higher than the pressure of 100 GPa for LaH_6_. LaTmH_12_ requires higher pressures to remain stable, probably due to the excessive gap between the properties of La and Tm. This type of ternary clathrate structure requires the two metal elements to be close in radius and other properties to ensure H cage stability. Thus, YTmH_12_ is able to stabilize at 80 GPa and exhibited *T*_*c*_ of 40–46 K. Both the minimum stabilization pressure and *T*_*c*_ are intermediate between the binary hydrides YH_6_ and TmH_6_. Yb and Lu, which are also heavy rare earth elements adjacent to Tm, have *f* electrons that can similarly enhance chemical pre-compression, so the stabilization pressure of their doped structures can be reduced even further, and YbTmH_12_ and LuTmH_12_ can be stabilized at 60 and 70 GPa, respectively, and exhibited *T*_*c*_ of 42–48 K. Their minimum stabilizing pressures and *T*_*c*_ also show a pattern intermediate to that of the binary hydrides.

Charge transfer has an important effect on the structure and properties of hydrides. Table 2 shows charges transferred for all thulium substituted clathrate hexahydrides. The *e* represents the total remaining electrons. Negative δ mean loss of electrons, positive δ mean gain of electrons. It can be seen that La is a very good electron donor and is able to provide sufficient electrons to the surrounding H. In LaTmH_12_, each La atom can provide 2.25 electrons, ultimately making 0.21 electrons available for each H on average. However, the provision of sufficient electrons does not necessarily mean that superconductivity is favored, and may even create factors that are detrimental to superconductivity. In terms of charge transfer, the ability of Tm to provide electrons is stronger than that of Y and Yb, but unfortunately, the presence of *f* electrons severely constrains higher *T*_*c*_ in thulium substituted clathrate hexahydrides.

**Table 2:** Charges transferred for compounds (a) YTmH_12_, (b) YbTmH_12_, (c) LuTmH_12_, (d) LaTmH_12_.

Compounds	Pressure (GPa)	*δ*(X)	*δ*(Tm)	*δ*(H)
YTmH_12_	80	− 1.09	− 1.19	0.19
YbTmH_12_	60	− 0.83	− 1.09	0.16
LuTmH_12_	70	0.94	− 2.54	0.13
LaTmH_12_	170	− 2.25	− 0.25	0.21
YH_6_	120	− 1.38	−	0.23
TmH_6_	50	–	− 0.91	0.15
YbH_6_	70	− 0.96	–	0.16
LuH_6_	100	− 0.66	–	0.11
LaH_6_	100	− 1.44	–	0.24

Negative δ mean loss of electrons, positive δ mean gain of electrons.

**Figure 4:**
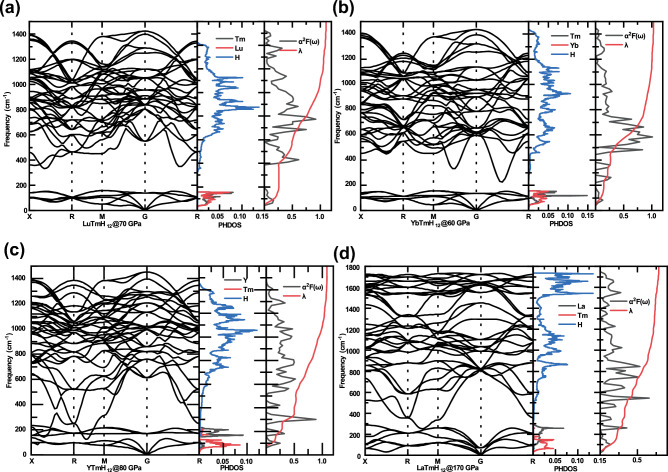
The calculated phonon band structure, PHDOS, electron–phonon coupling (EPC) parameter λ, and Eliashberg spectral function α^2^F(ω) of (**a**) LuTmH_12_, (**b**) YbTmH_12_, (**c**) YTmH_12_, (**d**) LaTmH_12_.

To determine the origin of the superconductivity in these superconductors, we calculated their phonon spectrum, projected phonon density of state (PHDOS), integral EPC parameter λ and Eliashberg spectral function α^2^F(ω). The superconductivity of superconductors comes mainly from strong electron–phonon coupling (EPC). So, we can look for the frequency range in which the EPC parameter λ grows rapidly, and vibration modes in this frequency range are the key to the superconductivity of this structure. As can be easily seen in Fig. 4, λ grows rapidly in two regions: the low-frequency region and the mid-frequency region. For example, in LuTmH_12_, λ grows rapidly to 0.25 in the frequency range of 0–150 cm^−1^ and then grows slowly until the frequency range of 400–1000 cm^−1^, where λ grows rapidly to 1.1, and then grows hardly at all (see Fig. 4a). In YbTmH_12_, λ also grows rapidly in the frequency range of 500–1000 cm^−1^ (see Fig. 4b). By comparing the PHDOS of different elements, we can find the reason for the rapid growth of λ. The rapid growth of λ is mainly due to the vibrations of metal atoms in the low-frequency region (red and black peaks in PHDOS), while in the mid-frequency region it is due to the vibrations of hydrogen atoms (blue peaks in PHDOS). This corresponds to the two main sources of superconductivity in such clathrate hydrides: hydrogen on the hydrogen cage and the central metallic atom. In addition to this, it can be seen in Fig. 4c that the λ of YTmH_12_ grows rapidly when the frequency is 300 cm^−1^. On the phonon dispersion, there are soft phonon patterns near the R direction in this frequency range. This suggests that the softening of the optical branch of the phonon spectrum is also an important source of electron–phonon coupling. In LuTmH_12_, λ grows rapidly to 0.25 in the frequency range of 0–150 cm^−1^ consistent with YTmH_12_ (see Fig. 4d). However, in higher frequency range, λ grows much slower than that in YTmH_12_, which leads to the low *T*_*c*_ in LaTmH_12_.

In this work, we introduce other elements to improve the superconductivity of TmH_6_, allowing the newly formed thulium substituted clathrate hexahydrides XTmH_12_ (X = Y, Yb, Lu and La) to have the higher *T*_*c*_ while maintaining low-pressure stability. Most prominently, YbTmH_12_ can be stabilized at a pressure of 60 GPa. Its *T*_*c*_ is elevated compared to the binary TmH_6_, reaching 48 K. The results provide an effective method for the successful design of hydride superconductors at moderate pressures.

## Computational methods

The candidate phases of XTmH_12_ (X = Y, Yb, Lu, and La) are predicted using the ab initio Random Structure search (AIRSS) technique^52,53^. The selected cut-off energy of the projected augmented wave (PAW)^54^ is 400 eV. The sampling density of the Brillouin district is 2π × 0.07 Å^−1^.The ultra-soft potentials is dynamically generated by the method of pseudo-potentials. The valence electrons in the electronic states of Y, Tm, Yb, Lu and La atoms are 4s^2^4p^6^5s^2^4d^1^, 4f^13^5s^2^5p^6^6s^2^, 4f^14^5s^2^5p^6^6s^2^, 4f^14^5p^6^5d^1^6s^2^, 5s^2^5p^6^5d^1^6s^2^, respectively.

Structural relaxation, calculations of enthalpies, band structures, density of states and charge transfer of XTmH_12_ (X = Y, Yb, Lu and La) at different pressures were calculated by the Cambridge Serial Total Energy Package (CASTEP)^55^. We use the generalized gradient approximation (GGA)^56^ with the Perdew-Burke-Ernzerh of (PBE) parametrization^57^ as the exchange–correlation function. For the plane wave, we chose a cut-off energy of 800 eV. The sampling density of the Brillouin region is 2π × 0.03 Å^−1^. The pseudo-potential is dynamically generated by the ultra-soft potential.

Phonon dispersion, electron–phonon coupling and Eliashberg spectral function α^2^F(ω) of XTmH_12_ (X = Y, Yb, Lu and La) were calculated by the Quantum-ESPRESSO (Open-Source Package for Research in Electronic Structure, Simulation, and Optimization)^58^. With an ultra-soft potential and a cut off energy of 90 Ry, all XTmH_12_ (X = Y, Yb, Lu, La) in the first Brillouin region have a k-point grid of 12 × 12 × 12 and a q-point grid of 4 × 4 × 4, respectively. The superconducting transition temperatures of XTmH_12_ (X = Y, Yb, Lu and La) are estimated through the Allen−Dynes-modified McMillan equation (A-D-M) with correction factors^51,59^:

$${T}_{c}=\frac{{f}_{1}{f}_{2}{\omega }_{log}}{1.2}exp\left[-\frac{1.04\left(1+\lambda \right)}{\lambda -{\mu }^{*}\left(1+0.62\lambda \right)}\right]$$

λ and ω_log_ are given by:

$$\lambda =2{\int }_{0}^{\infty }\frac{{\alpha }^{2}F(\omega )}{\omega }d\omega$$ and $${\omega }_{log}=exp\left(\frac{2}{\lambda }{\int }_{0}^{\infty }\frac{d\omega }{\omega }{\alpha }^{2}F(\omega )\mathit{ln}\omega \right)$$

*f*_1_ and *f*_2_ are given by:

$${f}_{1}=\sqrt[3]{\left[1+{\left(\frac{\lambda }{2.46(1+3.8{\mu }^{*})}\right)}^\frac{3}{2}\right]}$$ and $${f}_{2}=1+\frac{\left(\frac{{\omega }_{2}}{{\omega }_{log}}-1\right){\lambda }^{2}}{{\lambda }^{2}+\left[1.82(1+6.3{\mu }^{*})\frac{{\overline{\omega }}_{2}}{{\omega }_{log}}\right]}$$

average frequencies ${\overline{\omega }}_{2}$ is given by:

$${\overline{\omega }}_{2}=\sqrt{\frac{2}{\lambda }}{\int }_{0}^{\infty }\frac{d\omega }{\omega }{\alpha }^{2}F(\omega )\omega d\omega$$

The typical value of Coulomb pseudo-potential μ* was set as 0.1–0.13.

### Supplementary Information

Supplementary Figure S1.
