# Supplementary material for: Superconductivity of thulium substituted clathrate hexahydrides at moderate pressure
Source: Sci Rep. 2024 May 10;14:10729. doi: 10.1038/s41598-024-61400-z (PMC11087549; doi:10.1038/s41598-024-61400-z)
Supplement: Supplementary file 1 — Supplementary Figure S1. [file 41598_2024_61400_MOESM1_ESM.docx]

# Supplementary Material

# Superconductivity of Thulium Substituted Clathrate Hexahydrides at Moderate Pressure

Hongyu Huang^1^, Chao Deng^1^, Hao Song^1^, Mingyang Du^1,^*, Defang Duan^2^, Yanhui Liu^1^, Tian Cui^1,2,^*

*^1^ Institute of High Pressure Physics, School of Physical Science and Technology, Ningbo University, Ningbo, 315211, People's Republic of China.*

*^2^ College of Physics, Jilin University, Changchun 130012, People's Republic of China.*

** Corresponding author: dumingyang@nbu.edu.cn,* *cuitian@nbu.edu.cn (C. Author).*


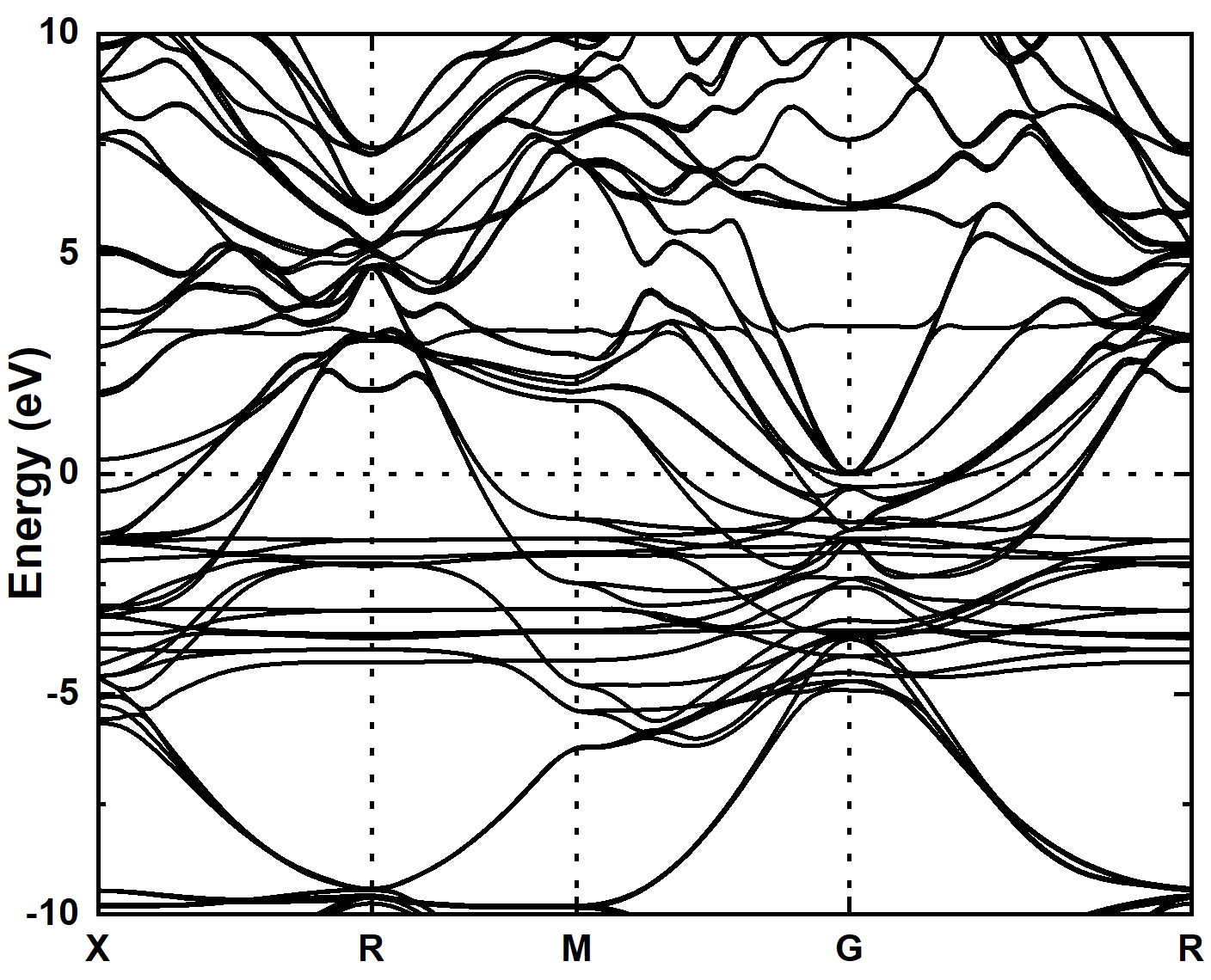


Fig. S1 Calculated electronic band structures and projected density of states with DFT+U (5eV) for YTmH_12_.
